# Protocol for the development of a tool to map systemic sclerosis pain sources, patterns, and management experiences: a Scleroderma Patient-centered Intervention Network patient-researcher partnership

**DOI:** 10.1186/s41927-024-00398-3

**Published:** 2024-06-21

**Authors:** Tiffany Dal Santo, Meira Golberg, Elsa-Lynn Nassar, Marie-Eve Carrier, Sophie Hu, Linda Kwakkenbos, Susan J. Bartlett, Rina S. Fox, Yvonne C. Lee, John Varga, Andrea Benedetti, Brett D. Thombs, the Scleroderma Patient-centered Intervention Network Pain Patient Advisory Team, Jo-Ann Lapointe McKenzie, Amanda Lawrie-Jones, Tracy Mieszczak, Silvia Petrozza, Maureen Sauve, Gayle Wixson

**Affiliations:** 1 Lady Davis Institute for Medical Research, Jewish General Hospital, Montreal, QC Canada; 2 Department of Psychiatry, McGill University, Montreal, QC Canada; 3 Department of Psychology, McGill University, Montreal, QC Canada; 4 Department of Epidemiology, Biostatistics, and Occupational Health, McGill University, Montreal, QC Canada; 5 Department of Clinical Psychology, Behavioural Science Institute, Radboud University, Nijmegen, the Netherlands; 6 Department of IQ Health, Radboud University Medical Center, Nijmegen, Netherlands; 7 Centre for Mindfulness, Department of Psychiatry, Radboud University Medical Center, Nijmegen, the Netherlands; 8 Department of Medicine, McGill University, Montreal, QC Canada; 9 College of Nursing, University of Arizona, Tucson, AZ USA; 10 Department of Medicine (Rheumatology) and Preventive Medicine, Northwestern University, Evanston, IL USA; 11 Department of Internal Medicine, Division of Rheumatology), University of Michigan, Ann Arbor, Michigan, USA; 12 Research Institute of the McGill University Health Centre, Montreal, QC Canada; 13 Respiratory Epidemiology and Clinical Research Unit, McGill University Health Centre, Montreal, QC Canada; 14 Biomedical Ethics Unit, McGill University, Montreal, QC Canada

**Keywords:** Cohort, KAMILA, Latent profile analysis, Nominal group technique, Pain, Qualitative methods, Systemic sclerosis

## Abstract

**Introduction:**

Systemic sclerosis (SSc) is a rare, complex autoimmune rheumatic disease with multiple factors that contribute to pain. People with SSc emphasize the effect pain has on their quality of life, but no studies have systematically examined the frequency and relative importance of different SSc pain sources, patterns of pain from different sources, and pain management experiences. Our objectives are to (1) develop a tool, jointly with researchers, health care providers, and patients, to map sources of pain in SSc, determine patterns of pain from different sources, and understand pain management experiences; and (2) administer the final tool version to participants in the large multinational Scleroderma Patient-centered Intervention Network (SPIN) Cohort.

**Methods:**

First, we will use validated pain assessment tools as templates to develop an initial version of our pain assessment tool, and we will obtain input from patient advisors to adapt it for SSc. The tool will include questions on pain sources, pain patterns, pain intensity, pain management techniques, and barriers to pain management in SSc. Second, we will conduct nominal group technique sessions with people living with SSc and health care providers who care for people with SSc to further refine the tool. Third, we will conduct individual usability testing sessions with SPIN Cohort participants. Once the tool has been finalized, we will administer it to individuals in the multinational SPIN Cohort, which currently includes over 1,300 active participants from 54 sites in 7 countries. We will perform unsupervised clustering using the KAy-Means for MIxed LArge data (KAMILA) method to identify participant subgroups with similar profiles of pain sources (present or absent) and to evaluate predictors of subgroup membership. We will use latent profile analysis to identify subgroups of participants with similar profiles based on pain intensity scores for each pain source and evaluate predictors.

**Discussion:**

Once completed, our pain assessment tool will allow our team and other researchers to map sources of pain in SSc and to understand pain management experiences of people living with SSc. This knowledge will provide avenues for studies on the pathophysiology of pain in SSc and studies of interventions to improve pain management.

**Supplementary Information:**

The online version contains supplementary material available at 10.1186/s41927-024-00398-3.

## Background

Systemic sclerosis (SSc, scleroderma) is a rare, chronic, autoimmune disease characterized by thickening and fibrosis of the skin and involvement of internal organs [1, 2]. Disease presentation is heterogeneous, and course is unpredictable [1, 2]. Common symptoms that impact the ability to carry out daily activities include hand function and mobility limitations, difficulty breathing, fatigue, gastrointestinal symptoms, and pain [3–11].

Many people with inflammatory rheumatic diseases live with persistent pain [12, 13], but the extent to which research and clinical care focus on pain varies across diseases [14]. Pain is a primary focus in rheumatoid arthritis, for example, but it is largely ignored in SSc [15, 16], despite being critically important to many people with SSc [17]. A recent study of over 2,000 participants with SSc from seven countries found that 38% reported moderate or severe pain, defined as a score ≥ 5 on a 0 to 10 scale. Mean pain intensity was 3.5 out of 10 [18], which is similar to levels observed in rheumatoid arthritis [18–20]. In a 2018 survey, pain management was identified as a top intervention research priority by people with SSc (*N* = 100), although none of the SSc health care providers who completed the same survey (*N* = 24) identified pain as a priority [17]. Moreover, pain is by far the most common reason people with SSc seek physical or occupational therapy [21]. Despite this, pain has not been the primary outcome of any SSc clinical trials, and few trials include pain as an outcome at all [18]. Major reviews of SSc pathology, disease manifestations, and clinical management only briefly mention pain [15, 16].

SSc is a complex disorder and multiple factors may contribute to pain, including skin and joint inflammation, Raynaud’s phenomenon, digital ulcers, calcinosis, joint contractures, gastrointestinal manifestations, and tendon friction rubs, as well as overlapping conditions such as rheumatoid arthritis and Sjögren’s syndrome [18, 22, 23]. To understand pain in SSc, it is important to assess both sources of pain and the characteristics of pain from each source. However, most existing pain assessments [24–26] are global pain scales that generate overall pain intensity or interference scores or domain scores, such as sensory and affective aspects of pain [25]. These measures do not provide an in-depth roadmap to the pain experience from each of multiple possible sources, nor do they address features of pain that are critical in SSc. To date, no studies have examined the prevalence or relative importance of different SSc pain sources, evaluated patterns of pain from different sources (e.g., frequency, duration, chronic or episodic nature, fluctuations in intensity), or assessed patient experiences with pain management.

The aim of the proposed research is to develop, test, and administer a tool to map the pain experience of people with SSc. The specific objectives are to: [1] develop a tool, jointly with researchers, health care providers, and patient partners, to assess sources of pain in SSc, determine patterns of pain from different sources, and understand pain management experiences and barriers to pain management; and [2] administer the final version of the tool in the multinational Scleroderma Patient-centered Intervention Network (SPIN) Cohort [27–29].

## Methods

SPIN investigators will partner with a SPIN Pain Patient Advisory Team to develop a preliminary version of a pain assessment tool designed to map pain in SSc. We will then present this tool to people with SSc and health care providers who care for people with SSc from SPIN’s network to obtain feedback via nominal group technique (NGT) sessions [30] and individual usability testing sessions. The tool will be refined based on this feedback, and once the tool has been completed, we will administer the final version in the SPIN Cohort.

### Development of initial version of the scleroderma pain assessment tool

SPIN researchers will meet with a project-specific SPIN Pain Patient Advisory Team of six people with SSc who will review a list of possible sources of SSc pain. The list will be developed based on previous studies of SSc pain sources [18, 22, 31]. Members of the Patient Advisory Team will review the list and offer suggestions on sources in the list and any additional pain sources they believe are important and should be included in the tool, as well as characteristics of pain to capture from different sources (e.g., frequency; duration; words used to describe type of pain) [32–34].

To create the initial version of the tool, we will refer to the structure of an existing mapping tool, the Mainz Pain Staging System (MPSS) [35–37]. The MPSS is a multi-dimensional pain assessment tool developed for research and clinical management of general chronic pain. Pain dimensions assessed in the MPSS include persistence (e.g., frequency, duration, chronic or episodic nature, fluctuations in intensity), bodily distribution, and health care. We will adapt the tool based on Patient Advisory Team input and will include pain dimensions for all pain sources included in the final list. Once adapted, members of the Patient Advisory Team will provide additional input on the tool structure and items, including usability aspects and item wording.

### NGT sessions

The NGT is a consensus technique that is used to develop or review assessment tool items directly with stakeholders [30]. We have used it successfully in previous studies [38–40]. To ensure that our assessment tool captures the experiences of people with SSc, we will conduct four to six NGT sessions, each lasting 60–90 min, with four to eight people with SSc per session. The final number of NGT sessions will be determined based on the redundancy and consistency of data obtained. NGT sessions will be conducted separately, in English and French, via Zoom.

#### Eligibility and recruitment of NGT session participants

Eligible participants must have SSc as confirmed by a physician, be fluent in English or French, have a mean PROMIS-29 Version 2.0 Pain Interference domain score consistent with mild to severe pain interference (PROMIS-29 Pain Interference T-score ≥ 55), and have a PROMIS-29 Pain Intensity score ≥ 1 (numerical rating scale from 0 to 10) [24, 41, 42]. See Supplement 1 for descriptions of the PROMIS-19 Pain Intensity and Pain Interference measures. Participants will be recruited from the ongoing SPIN Cohort and externally via posts on SPIN’s X account and Facebook page and via SPIN patient organization partner member emails and social media posts. These posts will provide access to an online consent form which will also serve to assess their eligibility. If we do not recruit a sufficient number of participants via our social media posts, SPIN Cohort participants who complete measures in English or French will be sent an invitation email that provides them with details of the study and a link to an online consent form where their eligibility will be assessed. See Supplement 2 for English and French invitation emails.

Eligibility for the SPIN Cohort requires a diagnosis of SSc confirmed by a SPIN physician; age ≥ 18 years; fluency in English, French, or Spanish; and access to a device with Internet access. Cohort participants are recruited during regular medical visits at one of 54 SPIN recruitment sites in seven countries (Australia, Canada, France, Mexico, Spain, UK, USA), and written informed consent is obtained for cohort participation and to be contacted about future studies. A medical data form is submitted online by the recruiting site to enroll participants. Cohort participants are then sent an email to activate their account and invited to complete online measures upon enrolment and at three-month intervals.

#### Consent and Pre-NGT survey

The online consent form will be administered via *Qualtrics* and will include a detailed description of the study and the NGT process and statements regarding participation and discontinuation, including assurance that the study does not involve any serious risk and that participants can withdraw at any time without consequence. Individuals with questions about the study will be encouraged to contact the study coordinator by email or phone prior to providing consent. Individuals who consent will proceed to a questionnaire that includes PROMIS-29 Version 2.0 Pain Interference and Pain Intensity items to assess eligibility. Individuals outside the SPIN Cohort will also complete a sociodemographic and medical information survey with information on sex, gender, age, race or ethnicity, country, relationship status, educational attainment, occupational status, family household income, and medical information including SSc diagnosis subtype, years since SSc diagnosis, and experience with important sources of pain (e.g., ulcer pain, joint contractures, gastrointestinal pain). Individuals in the SPIN Cohort will be asked to provide the email address linked with their SPIN account to allow us to access their sociodemographic and medical information. These data will be extracted from their baseline SPIN Cohort assessment. All respondents will also provide their availabilities for NGT sessions. The survey will include a message that it is possible that not all eligible participants will be assigned to a group, depending on the number of participants and scheduling availability. See Supplements 3 and 4 for the English and French versions of the consent form version for external recruitment of participants not in the SPIN Cohort; the SPIN Cohort version is a shorter version of this.

The study coordinator will assign participants to sessions, if possible, based on their availability and attempting to form groups that are diverse based on sociodemographic and disease information. Invitation emails will then be sent to confirm the date and time of each participant’s session and to confirm participation. See Supplement 5 for English and French NGT session invitation emails. If necessary, a follow-up email will be sent a week after the initial email to potential participants who have not responded. When a participant confirms attendance for a session, they will receive via email an additional short survey to complete prior to the NGT session. This part of the survey is being done separately to avoid burdening potential participants who cannot be assigned to a session. See Supplements 6 and 7 for the English and French versions of the pre-NGT surveys.

In the first part of the pre-NGT survey, participants will be presented with a list of the sources of pain included in the initial version of the tool and will be asked to rate the importance of including each listed pain source in the final tool on a scale of 0 (not at all important) to 10 (extremely important). For each pain source, participants will also be instructed to check a box if they believe any revisions are warranted and provide a brief explanation for their recommendation. Participants will then be asked to list any pain sources that they believe are missing from the list. In the second part of the pre-NGT survey, participants will be presented with a set of items about each pain source, including questions on pain intensity, pain frequency, and pain management techniques. They will similarly be asked to rate the importance of each item and provide recommendations.

Prior to each NGT session, the study coordinator will email each participant with the lists they submitted of potential new pain sources, sources they believe should be removed, and sources they believe should be modified, as well as questions they believe should be removed or modified, so that they can share them during their NGT session. Participants will receive a reminder email informing them that (1) they may log into Zoom 15 min before the start of their session to allow the moderators to help them resolve any technical issues (2), they should have their lists available at the start of the session, and (3) they should have a writing utensil and paper (or alternative device) available for brainstorming items during the session. See Supplement 8 for the follow-up email in English and French.

#### Protocol for NGT sessions

Two bilingual moderators will guide the English- and French-language NGT sessions. The moderators will be team members who are knowledgeable about SSc and have experience with discussion-based research.

Each session will begin with an introduction and overview of the session by the moderators. Sessions will be conducted using a round-robin format. Participants will first take turns presenting their list of potential new pain sources, and the group will discuss the relevance of each. Next, participants will be presented with a list, prepared in advance by the study coordinator, of pain sources taken from the pre-NGT questionnaire that were rated poorly or had modification suggestions. Participants will then be given the opportunity to discuss their previous low ratings of these sources as well as the importance and relevance of each to SSc patients. Using the same discussion format, participants will share their input on the items regarding pain intensity, patterns, and pain management techniques.

Once the group has developed a final list of new pain sources and previously low-rated sources, as well as new and previously low-rated items on pain intensity, patterns and pain management techniques, a moderator will transfer the list into a ready-made survey template in *Qualtrics* to allow participants to evaluate the items. See Supplement 9 for the survey template in English and French. The survey link will be sent to participants via email before the end of the session. Participants will be given the option of completing the survey while still in the videoconference session to give them the opportunity to ask questions directly to the moderators if any aspect of the survey is unclear. They may also choose to complete the survey independently and submit it within 48 h of receiving the link. In the survey, participants will rate each item from 0 (not at all important) to 10 (extremely important) based on how important they perceive each item to be. Across sessions, as new items are proposed and previously low-rated items are reviewed, our pain mapping tool will be revised by team researchers, and the revised tool will be used as a template during the next NGT session.

In addition to patient sessions, we will plan to conduct two NGT sessions with experienced health care providers (total *N* = 8–12) who care for people with SSc from SPIN’s network using similar methods.

#### Individual usability testing protocol

To conduct usability testing, we will recruit diverse participants (e.g., disease characteristics, age, education level, working status, sex or gender, language, race or ethnicity) from the SPIN Cohort and externally. Participants will be recruited in the same manner as for the NGT sessions. Each participant will meet online with two researchers who will introduce the participant to the tool and take field notes. Participants will be instructed to review the tool and its items via a ‘Think Aloud’ process, a common strategy for patient tool usability testing [43–45]. Once participants complete their review, brief open-ended questions will be asked (“Would you change anything about the tool?” and “Do you have any other comments or suggestions for us?”). After each interview, the researchers will debrief and compare field notes; any discrepancies in notes and opinions will be resolved through discussion with a third researcher, if necessary. Usability testing session data will be analyzed using conventional content analysis [46] and adapted constant comparative analyses [46, 47]. In this way, data analysis will begin after the first usability session to inform subsequent sessions, and code categories and patterns will be refined as new data are obtained. Considering that the tool will be co-created with team members with lived experience, we anticipate that we will achieve saturation on usability input after six to eight participants; however, the sample size will depend on item consistency and redundancy. Findings will be reviewed by the research team, revisions will be made, and if major changes are required another usability cycle will ensue.

#### Administration in the SPIN Cohort

After usability testing, we will administer the final pain mapping tool to individuals in the SPIN Cohort. Given its length, individuals will be invited to complete the questionnaire outside of their regular assessments, which we have done successfully in previous studies on physical activity [48], nutrition [49], and COVID-19 vaccination [50]; these studies were each completed by 721 to 932 participants. We will email SPIN Cohort participants to describe the study and assessment tool and invite them to complete it.

### Data analysis

To assess results from the SPIN Cohort administration, we will describe SPIN Cohort participant characteristics of those who complete and do not complete the pain assessment tool. Then, for all pain sources, we will describe the prevalence, intensity, patterns, pain management techniques and barriers to pain management by pain source. To identify participant subgroups with similar pain source profiles, based on the presence or absence of each source, and to attempt to identify predictors of subgroup membership, we will perform unsupervised clustering using the KAy-Means for MIxed LArge data (KAMILA) method, which is a model-based discriminative method [51–53]. To identify subgroups of participants with similar pain source profiles based on 0–10 pain intensity scores, we will perform latent profile analysis [54].

#### KAMILA unsupervised clustering

The KAMILA clustering algorithm is a semi-parametric extension of K-means clustering that accommodates mixed variable types and, unlike other mixed-type clustering algorithms [51–53], does not require a user-specified relative weighting of continuous and categorical predictors [53]. Instead of relying on restrictive parametric distributional specifications for continuous predictors of cluster membership, the algorithm employs the kernel density estimation method to estimate the distribution of these predictors within clusters [53]. Categorical variables are modeled using a multivariate, multinomial distribution with dimension equal to the number of categorical variables under consideration, a specification which allows for the joint modeling of continuous and categorical predictors in the determination of cluster membership [55, 56].

In the cluster identification process, KAMILA calculates Euclidean distances between continuous variables and their nearest centroids using kernel density estimation, generating an estimated mixture distribution for the continuous variables. The algorithm initializes cluster assignments for individuals based on their relevant predictors and iteratively adjusts these assignments until the Euclidean distance between individuals’ continuous values and cluster centroids is minimized. Simultaneously, it aims to maximize the log probability of observing individuals’ combinations of categorical variables given their cluster memberships [53, 57]. Centroids and parameters are updated at each iteration to enhance the representation of clusters until clusters do not change between iterations. KAMILA has been shown to maintain high stability, efficiency, and overall performance compared to other unsupervised clustering methods when applied to clinical data, especially when dealing with predictors of mixed variable types [51, 52, 58, 59].

We will choose the optimal number of clusters using the prediction strength method, which assesses clustering stability through k-fold cross-validation and identifies the largest number of clusters that will result in a prediction strength above a chosen threshold across different subsets of the data; thresholds between 0.8 and 0.9 are often recommended with higher thresholds typically generating fewer clusters [60, 61]. Data will be fit with 20 initializations and 20 iterations per initialization to ensure stable results [57], though the algorithm will stop earlier should the clusters remain unchanged after any iteration.

To evaluate the validity and reliability of identified clusters, the Jaccard coefficient, which measures the similarity of two subsets on a cluster based on their cluster classification, will be utilized [61, 62]. Using bootstrap sampling, subsets of the data will be randomly selected, and KAMILA will be applied to each subset. In each bootstrapped clustering, Jaccard coefficients for each cluster will be computed, and the maximum value will be retained. Should the original clustering perform acceptably, we would expect a mean Jaccard score of at least 0.5 [63].

For cluster identification in the present study, we will use the binary status of each pain source for each participant to allow us to identify patterns of pain sources within clusters. We will consider relevant sociodemographic and non-pain SSc characteristics, which were selected based on prior research [18], as predictors of cluster membership, including age, sex, years since onset of first non-Raynaud’s symptoms, SSc subtype, interstitial lung disease, pulmonary arterial hypertension, history of SSc renal crisis, overlap syndromes (systemic lupus erythematosus, rheumatoid arthritis, Sjögren’s syndrome, autoimmune thyroid disease, idiopathic inflammatory myositis, primary biliary cirrhosis), and SSc-related antibodies (antinuclear antibodies, anti-centromere, anti-topoisomerase I, anti-RNA polymerase III). Prior to clustering, continuous predictors will be standardized to standard normal distributions. Predictors will not be weighted by importance in the clustering procedure, as the KAMILA algorithm balances the contributions of continuous and categorical variables within the clustering process [52, 53, 57].

KAMILA clustering, cross-validation and performance evaluation will be conducted in R version 4.2.1, RStudio Version 2022.7.1.554.

#### Latent Profile Analysis

Latent profile analysis is used to identify latent groupings based on individual patterns of continuous variables [54]. The approach assumes that individuals belonging to the same latent profile are similar such that their observed scores on continuous observed variables are from the same probability distribution. It also assumes that observations are independent [54] and that everyone in the population belongs to exactly one identified latent profile [64, 65]. Once a desired number of subgroups and observed variables to be considered in profiling are specified, expectation maximization is used to determine profile characteristics and assign individuals. The algorithm iteratively computes probability estimates that an individual belongs to each profile given their observed variables and assigns them to the one with the highest probability. Following this, observed parameter estimates are iteratively updated to maximize the likelihood of the observed data [65, 66].

We will test models with different numbers of latent profile classes to evaluate the ideal number of classes that should be included. Each of these models will include pain intensity scores for each source in the pain assessment tool. Sources for which participants do not experience pain will be assigned a pain intensity score of 0. We will refer to the Akaike Information Criteria (AIC), Bayesian Information Criteria (BIC), Bootstrapped Likelihood Ratio Test (BLRT), sample size adjusted, Bayesian Information Criteria (ssBIC), and the Vuong-Lo-Mendell-Rubin likelihood ratio test (VLMR) model fit indicators to determine the optimal model and number of classes. We aim to select a model that has relatively low AIC, BIC, BLRT, ssBIC, and VLMR [67], entropy level ≥ 0.80 [68], and identifies clinically meaningful classes. We will exclude models with class sizes < 25 due to lack of parsimony and reduced power to identify predictors [54].

Once the model and classes have been selected, we will use multinomial logistic regression [69, 70] to identify predictors of class membership, using the same variables as in the KAMILA analysis. If findings are significant, we will perform post-hoc tests to get a better understanding of trend differences between each class.

We will use Mplus version 8.3 to conduct the latent profile analysis (Muthen & Muthen, Los Angeles, CA), and other analyses will be done with SAS version 9.4 (SAS Institute, Cary, NC).

### Sample size

Previous research has shown that a minimum sample size of 500 is enough to identify the correct number of latent profiles [54]. Given our large ongoing cohort, we expect to include over 1,000 participants.

## Data storage

All data collected during this study will be treated confidentially within the limits of the law. Only the NGT session moderators will have access to identifying participant information. All other investigators will only have access to participant code numbers. Access to identifying participant information will be restricted and supervised by Dr. Brett Thombs, the principal investigator. Data from NGT sessions and tool administration in the SPIN Cohort will be saved on McGill’s Dataverse repository indefinitely, where de-identified data will be stored securely on servers located in Canada. Data from the administration of the assessment tool to participants in the SPIN Cohort will be stored in a password-protected account in the online software program *Qualtrics.* After 10 years, only anonymized data will be retained on Dataverse. Anonymization will be done by removing all potentially identifying information from data files. After 10 years, all electronic files with any identifying information will be irreversibly deleted from hospital computers and from *Qualtrics*. Files will be deleted from peripheral devices in accordance with the best practices for each device.

## Risks and potential benefits

It is unlikely that there will be any harmful effects from the NGT sessions or usability testing. Participants will be given the opportunity to speak about their lived experiences with pain and participate in a consensus process on what items should be included in the assessment tool or to provide input on using the tool. It is, though, possible that some participants may experience discomfort or other negative emotions when discussing their illness, including discussion of symptoms and challenges. Participants will be informed that their participation is voluntary and that they may withdraw at any time. Should participants become distressed in any way, we will offer appropriate consultation. This study does not require clinical or laboratory tests. Although there are no anticipated immediate benefits, the information provided in these sessions will help advance SSc pain research, which we believe will stimulate research to improve clinical management of pain.

## Involvement of people with lived experience

People with lived experience who have SSc prioritized research on pain through their roles on SPIN’s Steering Committee. A six-member SPIN Pain Patient Advisory Team was involved in conceptualizing the study and will contribute to developing the initial version of the pain assessment tool, refining the tool based on feedback from others with lived experience, interpreting all study results, and disseminating study findings, including reviewing and co-authoring manuscripts that result from this study. Patient Advisory Team members reviewed the present protocol and are co-authors.

## Significance of the study

We will develop, jointly with patient partners and SPIN Cohort participants, a tool to assess sources of pain in SSc, patterns of pain from those different sources, current pain management services, and barriers to improving pain management. This is the first step to address key aspects of pain and pain management for people with SSc. The findings of this study will help us to better understand the multi-faceted nature of pain in SSc. This knowledge will provide avenues for studies on the pathophysiology of pain in SSc and studies of interventions to improve pain management, and the tool that we develop will be used by other researchers.

## Ethics and dissemination

This study has been approved as an amendment of the SPIN Cohort Study (#MP-05-2013-150) by the Research Ethics Committee of the Centre intégré universitaire de santé et de services sociaux du Centre-Ouest-de-l’Île-de-Montréal.

Study data will be shared with others via publication in a scientific journal, scientific and patient education conferences, invited speaker presentations, and academic posters. These reports will not include participants’ names or any information that could be used to identify them. General demographic information may be included to describe NGT and SPIN Cohort participants who complete the assessment tool.

## Study status

This is the first version of the protocol, finalized on April 3, 2024. No study participants have been recruited. Recruitment and enrollment are planned to begin in April 2024.

### Supplementary Information

Supplementary Material 1.

## Data Availability

All datasets used in this study will be archived through a McGill University repository (https://borealisdata.ca/dataverse/Thombs). Data will be made available upon request to the corresponding author and presentation of a methodologically sound proposal that is approved by the Scleroderma Patient-centered Intervention Network Data Access and Publications Committee. Data requesters will need to sign a data transfer agreement.

## Abbreviations

AIC: Akaike Information Criteria
BIC: Bayesian Information Criteria
BLRT: Bootstrapped Likelihood Ratio Test
KAMILA: KAy-Means for MIxed LArge data
MPSS: Mainz Pain Staging System
NGT: nominal group technique
SPIN: Scleroderma Patient-centered Intervention Network
ssBIC: sample size adjusted, Bayesian Information Criteria
SSc: Systemic sclerosis
VLMR: Vuong-Lo-Mendell-Rubin likelihood ratio test
