# Supplementary material for: Protocol for the development of a tool to map systemic sclerosis pain sources, patterns, and management experiences: a Scleroderma Patient-centered Intervention Network patient-researcher partnership
Source: BMC Rheumatol. 2024 Jun 21;8:28. doi: 10.1186/s41927-024-00398-3 (PMC11191384; doi:10.1186/s41927-024-00398-3)
Supplement: Supplementary file 1 — Supplementary Material 1. [file 41927_2024_398_MOESM1_ESM.docx]

**SUPPLEMENTAL MATERIAL**

**Supplement 1:** Descriptions of PROMIS-29 Pain Intensity and Pain Interference Measures

**Supplement 2:** Initial Invitation Emails

**Supplement 3:** English Consent Form

**Supplement 4:** French Consent Form

**Supplement 5:** NGT Invitation Emails

**Supplement 6:** English Pre-NGT Survey

**Supplement 7:** French Pre-NGT Survey

**Supplement 8:** Follow-up Emails

**Supplement 9:** Post-NGT Surveys

**Supplement 1:** Descriptions of PROMIS-29 Pain Intensity and Pain Interference Measures

Pain intensity and interference in the last 7 days will be evaluated with the Patient Reported Outcomes Information System (PROMIS)-29 profile version 2.0 (1). Pain intensity is assessed with a 11-point numeric rating item (0 = no pain to 10 = worst imaginable pain). Pain interference on daily functioning is assessed with 4 items (day to day activities; work around the home; participation in social activities; household chores), which are scored on a five-point response scale (1 = not at all to 5 = very much). Pain interference item scores are summed to yield a domain score, which is converted into a T-score calibrated to the United States general population (mean = 50, standard deviation [SD] = 10). The PROMIS-29 v2.0 has been validated in the SPIN Cohort (2). Pain interference scores can be classified as none (T-score < 50), mild (T-score 50-60), moderate (T-score 60-65), and severe (T-score > 65) (3).

**REFERENCES – Supplement 1**

1. Hays RD, Spritzer KL, Schalet BD, Cella D. PROMIS-29^®^ v2.0 profile physical and mental health summary scores. Qual Life Res. 2018;27:1885-91.
2. Kwakkenbos L, Thombs BD, Khanna D, Carrier ME, Baron M, Furst DE, et al. Performance of the Patient-Reported Outcomes Measurement Information System-29 in scleroderma: a Scleroderma Patient-centered Intervention Network Cohort Study. Rheumatology. 2017;56:1302-11.
3. Nagaraja V, Mara C, Khanna PP, Namas R, Young A, Fox DA, et al. Establishing clinical severity for PROMIS^®^ measures in adult patients with rheumatic diseases*.* Qual Life Res. 2018;27:755-64.

**Supplement 2: Initial Invitation Emails**

English Invitation Email

Subject: SPIN-PAIN: Invitation to take part in an online group discussion

Dear [NAME],

SPIN is seeking individuals living with scleroderma to participate in an online group discussion about their experiences with pain and how they receive adequate help for it. People living with scleroderma emphasize the impact of pain on their quality of life, but pain is often overlooked in research and clinical care. This study aims to develop a tool that will better assess different aspects of pain in scleroderma, including pain sources, patterns of pain, current pain management services, and barriers to improving pain management.

This online session will last 60-90 minutes and take place on the videoconferencing program *Zoom.* It will include other people living with scleroderma. In addition, you will be asked to complete some online questionnaires, prior to, and following the group session. You must have access to a computer or tablet with Internet to participate. No compensation will be offered.

Let us know if you are interested in participating in this study by completing the consent form and information sheet at [insert link]. We are happy to answer any questions via email (spinpainteam@gmail.com) or phone call (1-800-370-5099).

Kind regards,

The SPIN-PAIN Team

French Invitation Email

Objet : SPIN-PAIN (étude sur la douleur) : Invitation à participer à une discussion de groupe en ligne

Cher/chère [NOM],

SPIN recherche des personnes atteintes de sclérodermie pour participer à une discussion de groupe en ligne sur leur expérience de la douleur et sur la manière dont elles reçoivent une aide adéquate pour soulager cette douleur. Les personnes atteintes de sclérodermie soulignent l'impact de la douleur sur leur qualité de vie, mais la douleur est souvent négligée dans la recherche et les soins cliniques. Cette étude vise à développer un outil qui permettra de mieux évaluer les différents aspects de la douleur pour la sclérodermie, y compris les sources de douleur, les schémas de douleur, les services actuels de gestion de la douleur et les obstacles à l'amélioration de la gestion de la douleur.

Cette séance en ligne durera de 60 à 90 minutes et se déroulera sur la plateforme de vidéoconférence Zoom. D'autres personnes atteintes de sclérodermie y participeront. En outre, il vous sera demandé de répondre à des questionnaires en ligne, avant et après la séance de groupe. Vous devez avoir accès à un ordinateur ou à une tablette avec Internet pour participer. Aucune compensation ne sera offerte.

Faites-nous savoir si vous êtes intéressé(e) à participer à cette étude en remplissant le formulaire de consentement et la fiche d’information sur [lien inséré]. Nous nous ferons un plaisir de répondre à vos questions par courriel (spinpainteam@gmail.com) ou par téléphone 1-800-370-5099 (Canada), 01 86 26 53 61 (France).

Cordialement,

L'équipe SPIN-PAIN (étude sur la douleur)

**Supplement 3: English Consent Form**

**CONSENT TO PARTICIPATE IN**

A Scleroderma Patient-Centered Intervention Network Patient-Researcher Partnership to

Understand Pain Sources, Patterns, and Barriers to Effective Management in Systemic Sclerosis

Principal Investigator: Dr. Brett Thombs

1. **Introduction**

We are asking for your participation in a study in which we will conduct nominal group technique (NGT) sessions (structured group discussion) to assess sources of pain, patterns of pain from different sources, and current pain management services as well as barriers to improving pain management in scleroderma. We will use this information to develop a research tool that will thoroughly assess all aspects of pain in SSc. The long-term goal of the proposed research is to test and disseminate this pain assessment tool, which will ultimately improve our understanding of the multi-faceted nature of pain in SSc. Additionally, it will improve pain management in this population through the potential use of the tool as a better means of communication regarding pain between scleroderma patients and physicians. Before you decide to participate in this study, please take as much time as you need to read the following information carefully. Please let us know if there is anything that is not clear or if you would like more information about the study.

1. **What is the purpose of the study?**

No studies have examined the relative importance and commonality of different scleroderma pain sources, evaluated patterns of pain from different sources (e.g., frequency, duration, chronic or episodic nature, fluctuations in intensity), or assessed how to improve pain management. Thus, it is important to acquire more knowledge on these aspects in order to adequately determine how to help individuals in need. The purpose of this study is to learn more about the types of pain experienced by people living with scleroderma and details regarding their different experiences with this pain. Using information and patient input from these group discussions, we will be able to develop a tool to assess sources of pain in scleroderma, patterns of pain from those different sources, current pain management services, and barriers to improving pain management. During your scheduled group discussion, we will be asking you to tell us about your experiences with pain, as a person living with scleroderma, including your history with receiving help for your pain.

1. **Do I have to take part in the study?**

Whether or not you would like to take part in this study is entirely up to you. If you decide to

participate, you will be asked to sign an online consent form. You will also receive a copy of the

consent form via email. You are free to leave the study at any time and you do not need to give a reason.

1. **What will happen if I take part in the study?**

By signing the online consent form, you are agreeing to participate in one group discussion that will last 60-90 minutes. This session will take place online via the videoconferencing platform *Zoom.* A microphone is required to take part in this study. It is recommended that you use headphones or earphones that have a built-in microphone; however, it is also acceptable if you use the built-in microphone contained within your computer or tablet. Each group will be led by two moderators and will involve 4-8 participants.

If you consent to participate in this study, you will proceed to a scheduling survey, in which you will complete a standardized questionnaire that addresses pain, sociodemographic and medical questionnaires, and mark your availabilities for the online session. Once you have completed the survey, the study coordinator will email you to confirm the date and time of your designated session, and confirm your participation. Once you have accepted the invitation to participate on this date, we will send you another survey to complete prior to the session. This survey is comprised of the set of questions that will be covered during the group discussion. You will be presented with a list of pain sources and be asked to rate their importance, and provide an overall input on the list of pain sources presented. You will also be presented with a set of items about each pain source, including questions on pain intensity, pain frequency, and pain management techniques, and will be asked to rate each item on a scale of 0 (not at all important) to 10 (extremely important), as well as provide your overall feedback.

The moderators will ask questions to participants during the discussion. You are free to answer any questions you wish to respond to, and you can also choose to not answer any questions. The moderators will ask participants to take turns in presenting their list of potential new pain sources, and the group will then discuss the relevance and independently rate the importance of each new item on a scale of 0 (not at all important) to 10 (extremely important) until a consensus is reached. Next, participants will be presented with a list, prepared in advance by the study coordinator, of pain sources taken from the pre-session questionnaire that were rated poorly or that had modification suggestions. The group will then be given the opportunity to discuss their previous low ratings of these items as well as the importance and relevance of each item to scleroderma patients. Lastly, participants will be asked to discuss their ratings and suggestions for the additional items that were presented to them prior to the session.

Once the group has developed a final list of new pain sources and previously low-rated sources, as well as new and previously low-rated items on pain intensity, patterns and pain management techniques, a moderator will transfer the list into a ready-made survey template in Qualtrics to allow participants to evaluate the items. This survey will be sent to you via email, and you will have the option of completing the survey while still in the videoconference and ask questions to the moderator if any aspect of the survey is unclear. You may also choose to complete the survey independently and submit it within 48 hours of receiving the link. In the survey, you will rate each item from 0 (not at all important) to 10 (extremely important) based on how important you perceive each item to be.

1. **What are the possible benefits of taking part in this study?**

Although there are no immediate benefits, the information you will provide in this session will help advance scleroderma research and inform the development of an evidence-based pain assessment tool for people living with scleroderma. Additionally, you will be given the opportunity to speak about your experiences with pain in an open, safe, non-judgmental environment.

1. **What are the possible risks of taking part in this study?**

It is unlikely that there will be any harmful effects from participating in this study. At the beginning of the session, we will ask everyone to agree to treat all information shared as confidential, although we cannot guarantee it. It is also possible that you may experience discomfort or other negative emotions when discussing your illness, including discussion of symptoms and challenges. Should you experience discomfort or distress during any part of the study, study moderators or study investigators will be available to offer consultation after the focus group, or refer you to appropriate resources, if you wish. As mentioned previously, your participation is voluntary and you may withdraw at any time. In addition, this study does not require clinical or laboratory tests.

1. **Will my taking part in the study be kept confidential?**

Participants will be asked not to discuss anything they heard from others after the focus group is over. Only the session moderators will have access to identifying participant information. All other investigators will only have access to participant code numbers. Access to identifying participant information will be restricted and supervised by Dr. Brett Thombs, the principal investigator.

The information gained from the sessions will be saved on McGill’s Dataverse repository indefinitely, where data will be stored securely on servers located in Canada and will be kept private. Information will also be stored in a password-protected account in the online software program Qualtrics. After 10 years, only de-identified data will be retained on Dataverse. De-identification will be done by removing all identifying information from the data file (name, email, phone). After 10 years, all electronic files with identifying information will be irreversibly deleted from hospital computers and from Qualtrics. Files will be deleted from peripheral devices in accordance with the best practice for each device.

1. **Use and publication of results**

All reports will only include information about the group as a whole, as well as trends that emerge from analysis of the data collected from the group. The study data may be published or shared with others in scientific discussions. We will not include your name or any information that could be used to identify you in any scientific publications or communications that will follow this study.

1. **Will there be compensation for participating in this study?**

No compensation will be provided for participating in the study.

1. **Voluntary participation and withdrawal rights**

Your participation is entirely up to you. You have the right to refuse to participate or to withdraw from the study, without giving any reason, at any time, and without any consequences to you. In the event that you withdraw or you are withdrawn from the study, all information collected up until that point for the purpose of this study may be used in order to preserve the scientific integrity of the study.

1. **Contact for further information**

Thank you for taking the time to read the information about this study. If you have any questions or concerns now or at any time about this study, you can contact the study coordinator, Tiffany Dal Santo, at [spinpainteam@gmail.com](mailto:spinpainteam@gmail.com) or call 1-800-370-5099.

**If you have any questions or concerns regarding your safety and rights as a participant in the study, you can call 1-800-370-5099.**

- I have read and understand the above information.
- I have had the chance to ask questions. All my questions have been answered to my

satisfaction.

**Please check one to proceed**

Yes, I provide my consent to participate in this study.

No, I do not provide my consent to participate in this study.

[If the individual provides consent, they will move on to the following set of questions]

**The following questions comprise of a section of the Patient-Reported Outcomes Measurement Information System (PROMIS) instrument that addresses pain:**

[PROMIS Questions]

**What is your sex assigned at birth?**

Female

Male

Intersex/disorder of sexual development

**What gender do you identify as? (Please check all that apply)**

Woman

Man

Transgender woman

Transgender man

Non-binary

Two-spirit

Other

**What is your age?** [textbox]

**Indicate the country/region where you live**

Canada

United States

European Union/United Kingdom

Other

Please specify the country where you live [textbox]

**What is your racial or ethnic background? (Please check all that apply)**

White

Black

Asian

Arab

Latin American

Aboriginal (North American Indian, Metis or Inuit)

Other

Please specify your other racial or ethnic background that was not provided in the list

**What is your current relationship status?**

Single

Married or living as married

Separated

Divorced

Widowed

**How many years of education have you completed, starting with elementary/primary school, and including all levels of formal education?**

[textbox]

**What is your current occupational status? (Please check all that apply)**

Homemaker

Unemployed

Retired

On disability

On leave of absence

Part-time employed

Full-time employed

Part-time student

Full-time student

Other

Please specify your other occupational status that was not provided in the previous list [textbox]

**What is your scleroderma diagnosis?**

Limited SSc

Diffuse SSc

I don’t know

Other (Please specify)

**How many years has it been since you received your scleroderma diagnosis? Provide your best estimate if you are not sure.** [textbox]

**Indicate what sources you have experienced pain from. (Please check all that apply)**

Ulcers

Contractures

Raynaud’s phenomenon

Gastrointestinal symptoms

Oro-facial

Muscle pain

Other (Please specify, e.g., carpal tunnel syndrome, trigeminal neuralgia, chest pain)

**SCHEDULING**

**We will now ask you to provide your availabilities so that we can schedule the NGT session. Please begin by selecting your time zone.**

Pacific Time (PT), (Vancouver BC, Los Angeles CA)

Mountain Time (MT), (Edmonton AB, Phoenix AZ)

Central Time (CT), (Winnipeg MB, Chicago IL)

Eastern Time (ET), (Toronto ON, Albany NY)

Atlantic Time (AT), (Fredericton NB)

Newfoundland Time (NT), (St. John's NL)

Western European Time (UTC), (London, Lisbon)

Central European Time (UTC+1), (Paris, Berlin)

Other

Please specify your time zone that was not provided in the list. [textbox]

**Please check all the time slots when you will be available to participate in an group session.**

DATE 1 (TIME)

DATE 2 (TIME)

DATE 3 (TIME)

DATE 4 (TIME)

DATE 5 (TIME)

DATE 6 (TIME)

I am not available during any of these times.

**Thank you for taking the time to complete our survey. We appreciate your interest in participating. We will contact you to let you know the date and time of your NGT group session.**

**Please note: Due to a high response rate and limited spots, we cannot guarantee that every person who completes the survey will get assigned to a group. Whether you get assigned to a group will depend on the availabilities you have provided and the number of participants who have already been assigned to those time slots.**

**Thank you,**

**The SPIN-PAIN Team**

**Supplement 4: French Consent Form**

**CONSENTEMENT À PARTICIPER À**

Un partenariat patient-chercheur du Réseau d’Intervention centré sur le patient sclérodermique (SPIN) pour comprendre les sources de douleur, les schémas et les obstacles à une prise en charge efficace dans la sclérose systémique

Chercheur principal : Brett Thombs

**1. Introduction**

Nous sollicitons votre participation à une étude dans le cadre de laquelle nous organiserons des séances de groupe nominal (discussion de groupe structurée) afin d'évaluer les sources de douleur, les schémas de douleur provenant de différentes sources, les services actuels de prise en charge de la douleur, ainsi que les obstacles à l'amélioration de la prise en charge de la douleur pour la sclérodermie. Nous utiliserons ces informations pour développer un outil de recherche qui évaluera en profondeur tous les aspects de la douleur dans la sclérodermie. L'objectif à long terme de la recherche proposée est de tester et de diffuser cet outil d'évaluation de la douleur, qui améliorera notre compréhension de la nature multidimensionnelle de la douleur dans la sclérodermie. En outre, elle permettra d'améliorer la prise en charge de la douleur grâce à l'utilisation potentielle de cet outil comme meilleur moyen de communication sur la douleur entre les patient.e.s atteint.e.s de sclérodermie et les médecins. Avant de décider de participer à cette étude, veuillez prendre le temps nécessaire pour lire attentivement les informations suivantes. N'hésitez pas à nous faire savoir si quelque chose n'est pas clair ou si vous souhaitez obtenir plus d'informations sur l'étude.

**2. Quel est l'objectif de l'étude?**

Aucune étude n'a examiné l'importance relative et le caractère commun des différentes sources de douleur liées à la sclérodermie, ni évalué les caractéristiques de la douleur provenant de différentes sources (par exemple, la fréquence, la durée, la nature chronique ou épisodique, les fluctuations d'intensité), ou évalué la façon d'améliorer la prise en charge de la douleur. Il est donc important d'acquérir davantage de connaissances sur ces aspects afin de déterminer de manière adéquate comment aider les personnes qui en ont besoin. L'objectif de cette étude est d'en apprendre plus sur les types de douleurs ressenties par les personnes atteintes de sclérodermie et sur les détails de leurs différentes expériences de la douleur. Grâce aux informations et aux commentaires des patients issus de ces discussions de groupe, nous serons en mesure de développer un outil permettant d'évaluer les sources de douleur pour la sclérodermie, les schémas de douleur provenant de ces différentes sources, les services actuels de gestion de la douleur et les obstacles à l’amélioration de la gestion de la douleur. Au cours de la discussion de groupe prévue, nous vous demanderons de nous parler de votre expérience de la douleur, en tant que personne vivant avec la sclérodermie, y compris de vos antécédents en matière d’aide pour soulager votre douleur.

3. **Suis-je obligé.e de participer à l'étude?**

Vous êtes entièrement libre de participer ou non à cette étude. Si vous décidez de participer, il vous sera demandé de signer un formulaire de consentement en ligne. Vous recevrez également une copie du formulaire de consentement par courrier électronique. Vous êtes libre de quitter l'étude à tout moment et vous n'avez pas besoin d'en donner la raison.

**4. Que se passera-t-il si je participe à l'étude?**

En signant le formulaire de consentement en ligne, vous acceptez de participer à une discussion de groupe d'une durée de 60 à 90 minutes. Cette séance se déroulera en ligne via la plateforme de vidéoconférence Zoom. Un microphone est nécessaire pour participer à cette étude. Il est recommandé d'utiliser un casque ou des écouteurs avec microphone intégré, mais il est également possible d'utiliser le microphone intégré de votre ordinateur ou de votre tablette. Chaque groupe sera dirigé par deux modérateurs et comprendra de 4 à 8 participants.

Si vous acceptez de participer à cette étude, vous passerez à une enquête de programmation, dans laquelle vous remplirez un questionnaire standardisé sur la douleur, des questionnaires sociodémographiques et médicaux, et vous indiquerez vos disponibilités pour la session en ligne. Une fois l'enquête terminée, le coordinateur de l'étude vous enverra un courrier électronique pour confirmer la date et l'heure de la session désignée, ainsi que votre participation. Une fois que vous aurez accepté l'invitation à participer à cette date, nous vous enverrons un autre questionnaire à remplir avant la session. Cette enquête comprend la série de questions qui seront abordées au cours de la discussion de groupe. On vous présentera une liste de sources de douleur et on vous demandera d'évaluer leur importance et de donner votre avis général sur la liste des sources de douleur présentée. On vous présentera également une série de questions sur chaque source de douleur, notamment sur l'intensité et la fréquence de la douleur, ainsi que sur les techniques de gestion de la douleur et on vous demandera d'évaluer chaque élément sur une échelle de 0 (pas du tout important) à 10 (extrêmement important), ainsi que de donner votre avis général.

Les modérateurs poseront des questions aux participant.e.s pendant la discussion. Vous êtes libre de répondre à toutes les questions auxquelles vous souhaitez répondre, et vous pouvez également choisir de ne répondre à aucune question. Les modérateurs demanderont aux participant.e.s de présenter à tour de rôle leur liste de nouvelles sources potentielles de douleur, puis le groupe discutera de la pertinence et évaluera indépendamment l'importance de chaque nouvel élément sur une échelle de 0 (pas du tout important) à 10 (extrêmement important) jusqu'à ce qu'un consensus soit atteint. Ensuite, les participant.e.s se verront présenter une liste, préparée à l'avance par la coordonnatrice de l'étude, comprenant les sources de douleur extraites du questionnaire pré-séance, qui ont été mal évaluées ou qui ont fait l'objet de suggestions de modification. Le groupe aura alors l'occasion de discuter des mauvaises notes qu'ils ont attribuées à ces éléments ainsi que de l'importance et de la pertinence de chaque élément pour les personnes atteintes de sclérodermie. Enfin, les participant.e.s seront invité.e.s à discuter de leurs évaluations et de leurs suggestions concernant les éléments supplémentaires qui leur ont été présentées avant la séance.

Une fois que le groupe a dressé une liste définitive des nouvelles sources de douleur et des sources précédemment mal évaluées, ainsi que des éléments nouveaux et précédemment mal évalués concernant l'intensité de la douleur, les schémas et les techniques de gestion de la douleur, un modérateur transférera la liste dans un modèle de sondage prêt à l'emploi Qualtrics afin de permettre aux participant.e.s d'évaluer les éléments. Un lien vers ce sondage vous sera envoyé par courriel et vous aurez la possibilité de le compléter pendant la séance et de poser des questions au modérateur si un aspect de l'enquête n'est pas clair. Vous pouvez également choisir de répondre au sondage de manière indépendante et de le soumettre dans les 48 heures suivant la réception du lien. Dans le sondage, vous noterez chaque élément de 0 (pas du tout important) à 10 (extrêmement important) en fonction de l'importance que vous lui accordez.

**5. Quels sont les avantages éventuels d'une participation à cette étude ?**

Bien qu'il n'y ait pas d'avantages immédiats, les informations que vous fournirez au cours de cette séance contribueront à faire avancer la recherche sur la sclérodermie et à informer le développement d'un outil d'évaluation de la douleur basé sur des données probantes pour les personnes atteintes de sclérodermie. De plus, vous aurez l'occasion de parler de votre expérience de la douleur dans un environnement ouvert, sécuritaire et sans jugement.

**6. Quels sont les risques éventuels liés à la participation à cette étude ?**

Il est peu probable que la participation à cette étude ait des effets néfastes. Au début de la séance, nous demanderons à chacun de garder confidentielles toutes les informations partagées, bien que nous ne puissions pas le garantir. Il est également possible que vous ressentiez une gêne ou d'autres émotions négatives lorsque vous discuterez de votre maladie, y compris de vos symptômes et de vos difficultés. Si vous ressentez un malaise ou une détresse pendant une partie de l'étude, les modérateurs ou les chercheurs de l'étude seront disponibles pour vous offrir une consultation après la discussion de groupe, ou vous orienter vers des ressources appropriées, si vous le souhaitez. Tel qu’indiqué précédemment, votre participation est volontaire et vous pouvez vous retirer à tout moment. En outre, cette étude ne nécessite pas de tests cliniques ou de laboratoire.

**7. Ma participation à l'étude restera-t-elle confidentielle ?**

Il sera demandé aux participant.e.s de ne pas discuter de ce qu'ils ont entendu de la part d'autres personnes après la discussion de groupe. Seuls les modérateurs de la séance auront accès aux informations permettant d'identifier les participant.e.s. Toutes les autres personnes impliquées dans l’étude n'auront accès qu'aux numéros de code des participant.e.s. L'accès aux informations d'identification des participant.e.s sera limité et supervisé par le Dr Brett Thombs, le chercheur principal.

Toutes les données recueillies dans le cadre de cette étude seront sauvegardées indéfiniment dans le dépôt Dataverse de McGill, où les données seront stockées en toute sécurité sur des serveurs situés au Canada et resteront confidentielles. Les informations seront également stockées dans un compte protégé par mot de passe dans le logiciel en ligne Qualtrics. Après 10 ans, seules les données dépersonnalisées seront conservées sur Dataverse. La dépersonnalisation sera effectuée en supprimant toutes les informations d'identification du fichier de données (nom, courriel, téléphone). Après 10 ans, tous les fichiers électroniques contenant des informations d'identification seront irréversiblement supprimés des ordinateurs de l'hôpital et de Qualtrics. Les fichiers seront supprimés des périphériques conformément aux meilleures pratiques en vigueur pour chacun d'entre eux.

**8. Utilisation et publication des résultats**

Tous les rapports comprendront seulement des informations sur le groupe dans son ensemble, ainsi que les tendances qui ressortent de l'analyse des données collectées auprès du groupe. Les données de l'étude pourront être publiées ou partagées avec d'autres dans le cadre de discussions scientifiques. Nous n'inclurons pas votre nom ni aucune information qui pourrait être utilisée pour vous identifier dans les publications scientifiques ou les communications qui suivront cette étude.

**9. Y aura-t-il une compensation pour la participation à cette étude?**

Aucune compensation ne sera accordée pour la participation à l'étude.

**10. Participation volontaire et droit de retrait**

Votre participation dépend entièrement de vous. Vous avez le droit de refuser de participer ou de vous retirer de l'étude, sans donner de raison, à tout moment et sans aucune conséquence pour vous. Dans le cas où vous vous retirez ou êtes retiré.e de l'étude, toutes les informations recueillies jusqu’au moment de votre retrait pourront être utilisées afin de préserver l'intégrité scientifique de l'étude.

**11. Contact pour de plus amples informations**

Nous vous remercions d'avoir pris le temps de lire les informations relatives à cette étude. Si vous avez des questions ou des inquiétudes, maintenant ou à tout moment, concernant cette étude, vous pouvez contacter la coordonnatrice de l'étude, Tiffany Dal Santo, à l'adresse spinpainteam@gmail.com ou en appelant au 1-800-370-5099.

**Si vous avez des questions ou des préoccupations concernant votre sécurité et vos droits en tant que participant.e à l'étude, vous pouvez appeler 1-800-370-5099.**

- J'ai lu et compris les informations ci-dessus.
- J'ai eu l'occasion de poser des questions. Toutes mes questions ont reçu des réponses satisfaisantes.

**Veuillez cocher une case pour continuer**

Oui, je consens à participer à cette étude.

Non, je ne consens pas à participer à cette étude.

[If the individual provides consent, they will move on to the following set of questions]

**Les questions suivantes font partie d'une section de l'instrument PROMIS (Patient-Reported Outcomes Measurement Information System) qui traite de la douleur:**

[Question PROMIS]

**Quel est votre sexe attribué à la naissance?**

Femme

Homme

Intersexe/troubles du développement sexuel

**À quel genre vous identifiez-vous? (Veuillez cocher toutes les cases qui s’appliquent)**

Femme

Homme

Femme transgenre

Homme transgenre

Non-binaire

Bi-spirituel

Autre

**Quel est votre âge?** [zone de texte]

**Indiquez le pays/la région où vous vivez**

Canada

États-Unis d'Amérique

Union européenne/Royaume-Uni

Autres pays

Veuillez préciser le pays dans lequel vous vivez [zone de texte]

**Quelle est votre origine raciale ou ethnique?** (Veuillez cocher toutes les cases qui s'appliquent)

Blanc.che

Noir.e

Asiatique

Arabe

Latino-américain.e

Autochtone (Indien d'Amérique du Nord, Métis ou Inuit)

Autre

Veuillez préciser vos autres origines raciales ou ethniques qui n'ont pas été mentionnées dans la liste.

**Quel est votre statut conjugal actuel?**

Célibataire

Marié.e ou vivant en union de fait

Séparé.e

Divorcé.e

Veuf.ve

**Combien d'années d'études avez-vous suivies, en commençant par l'école élémentaire/primaire, y compris tous les niveaux d'éducation formelle?**

[textbox]

**Quelle est votre situation professionnelle actuelle?** (Veuillez cocher toutes les cases qui s'appliquent)

Personne au foyer

Sans emploi

Retraité.e

En invalidité

En congé

Employé.e à temps partiel

Employé.e à temps plein

Étudiant.e à temps partiel

Étudiant.e à temps plein

Autre

Veuillez préciser votre autre situation professionnelle qui n'a pas été mentionnée dans la liste précédente [zone de texte]

**Quel est votre diagnostic de sclérodermie?**

Sclérodermie limitée

Sclérodermie diffuse

Je ne sais pas

Autre (Veuillez préciser)

**Combien d'années se sont écoulées depuis que vous avez reçu votre diagnostic de sclérodermie?** **Donnez votre meilleure estimation si vous n'êtes pas certain.e.** [zone de texte]

**Indiquez les sources de douleur que vous avez ressentis.es.** (Veuillez cocher toutes les réponses qui s'appliquent)

Ulcères

Contractures

Phénomène de Raynaud

Symptômes gastro-intestinaux

Oro-facial

Douleurs musculaires

Autres symptômes (Veuillez préciser, e.g., syndrome du canal carpien, névralgie du trijumeau, douleur thoracique)

**HORAIRES**

**Nous allons maintenant vous demander de nous indiquer vos disponibilités afin que nous puissions programmer la discussion de groupe. Commencez par sélectionner votre fuseau horaire.**

Heure du Pacifique (PT), (Vancouver BC, Los Angeles CA)

Heure des montagnes (MT), (Edmonton AB, Phoenix AZ)

Heure centrale (CT), (Winnipeg MB, Chicago IL)

Heure de l'Est (ET), (Toronto ON, Albany NY)

Heure de l'Atlantique (AT), (Fredericton NB)

Heure de Terre-Neuve (NT), (St. John's NL)

Heure de l'Europe occidentale (UTC), (Londres, Lisbonne)

Heure d'Europe centrale (UTC+1), (Paris, Berlin)

Autre

Veuillez préciser le fuseau horaire qui ne figure pas dans la liste. [zone de texte]

**Veuillez cocher toutes les plages horaires pour lesquelles vous êtes disponible pour participer à une séance de groupe.**

DATE 1 (HEURE)

DATE 2 (HEURE)

DATE 3 (HEURE)

DATE 4 (HEURE)

DATE 5 (HEURE)

DATE 6 (HEURE)

Je ne suis pas disponible à ces moments.

**Nous vous remercions d'avoir pris le temps de répondre à notre enquête. Nous apprécions l'intérêt que vous portez à l’étude. Nous vous contacterons pour vous communiquer la date et l'heure de votre séance de groupe.**

**Remarque: En raison d'un taux de réponse élevé et d'un nombre de places limité, nous ne pouvons pas garantir que toutes les personnes qui répondent à l'enquête seront assignées à un groupe. Votre affectation à un groupe dépendra des disponibilités que vous avez indiquées et du nombre de participant.e.s déjà affecté.e.s à ces plages horaires.**

**Nous vous remercions,**

**L'équipe SPIN-PAIN (étude sur la douleur)**

**Supplement 5: NGT Invitation Emails**

English Invitation Email

Subject: SPIN-PAIN Group Assignment

Dear [NAME],

Thank you for completing the survey to participate in the SPIN Pain Project. Based on the availabilities you have provided, we have scheduled you for a nominal group session on Month Date Year at TIME [XX:XX, XX AM/PM] TIME ZONE.

Please confirm by responding to this email that you are available to attend this session.

The session will take place on the online videoconference program, *Zoom*. Once you have confirmed your attendance, instructions on how to use this program will be sent to you, along with a final online survey to complete before your session.

Kind regards,

The SPIN-PAIN Team

French Invitation Email

Objet: Assignation de groupe SPIN-PAIN (étude sur la douleur)

Cher/chère [NOM],

Nous vous remercions d'avoir répondu à l'enquête pour participer au projet SPIN Pain – Étude sur la douleur. Sur la base des disponibilités que vous avez fournies, nous vous avons assigné à une séance de discussion de groupe le Mois Date Année à l'HEURE [XX:XX, XX AM/PM] ZONE HORAIRE.

Veuillez confirmer, en répondant à ce courriel, que vous êtes disponible pour participer à cette séance.

La discussion se déroulera sur la plateforme de vidéoconférence en ligne Zoom. Une fois que vous aurez confirmé votre participation, des instructions sur l'utilisation de ce programme vous seront envoyées, ainsi qu'un dernier sondage en ligne à remplir avant la session.

Cordialement,

L'équipe SPIN-PAIN (étude sur la douleur)

**Supplement 6: English Pre-NGT Survey**

**Initial Survey – Study on Pain Sources in Scleroderma**

**Rate the importance of each of the pain sources listed below on a scale of 0 (not at all important) to 10 (extremely important).**

**Then, if relevant, indicate if you believe this pain source should be modified and explain why (e.g., the description of the pain source is not clear or difficult to understand, the pain source is difficult to differentiate from the others).**

**Pain Source Modify (If yes, explain why)** [yes, no]

**Ulcers on fingers or toes**

[scale from 0 to 10]

**Ulcers in mouth or nose**

[scale from 0 to 10]

**Other ulcers**

[scale from 0 to 10]

**Gangrene in fingers or toes (lack of blood flow causing death of body tissue which becomes discolored or black)**

[scale from 0 to 10]

**Amputation (related to autoamputation)**

[scale from 0 to 10]

**Phantom pain (due to lost finger or toe)**

[scale from 0 to 10]

**Contractures of small joints (fingers, toes)**

[scale from 0 to 10]

**Contractures of large joints (knees, hips, elbows, shoulders)**

[scale from 0 to 10]

**Raynaud’s phenomenon**

[scale from 0 to 10]

**Tight, thick or hard skin**

[scale from 0 to 10]

**Calcinosis (deposition of insoluble calcium salts in the cutaneous and subcutaneous tissue, usually appearing as lumps under the skin)**

[scale from 0 to 10]

**Carpal tunnel syndrome (Neurological disorder involving the nerve which runs from your forearm into the palm of the hand. Symptoms include numbness, swollen fingers, tingling, loss of movement.)**

[scale from 0 to 10]

**Trigeminal neuralgia (sudden and severe sharp or shooting facial pain)**

[scale from 0 to 10]

**Gastrointestinal symptoms**

**If yes:**

- **Swallowing**
- **Nausea**
- **Acid reflux or heartburn**
- **Bloating**
- **Constipation**
- **Diarrhea**

[scale from 0 to 10]

**Oro-facial**

**If yes:**

- **Reduced mouth opening**
- **Dryness from reduced saliva production**
- **Bone resorption or erosion**
- **Periodontal diseases’ indices (tissue loss around the teeth, inflammation of the gums)**

[scale from 0 to 10]

**Muscle pain**

[scale from 0 to 10]

**Chest pain**

[scale from 0 to 10]

**Pain from sources other than scleroderma**

**If yes:**

- **Osteoarthritis**
- **Back pain**
- **Headaches**
- **Migraines**
- **Fibromyalgia (chronic disorder that causes pain and tenderness throughout the body, fatigue, and trouble sleeping)**
- **Other [textbox]**

[scale from 0 to 10]

**Do you believe there are any important pain sources missing in the list above? For example, is there a type of pain that you experience that has not been listed?**

[yes, no]

If yes, describe the source of pain

**Rate each set of questions listed below on a scale of 0 (not at all important) to 10 (extremely important).**

**Then, if relevant, indicate if you believe the question or answer options should be modified and explain why (e.g., the question or answer options are not clear, understandable, or relevant for scleroderma).**

**Questions and answers [scale 0 to 10] Modify? (If yes, explain why)** [yes, no]

**1. Since your first experience with [enter pain source], new episodes have been:**

- Infrequent (typically more than 3 months between the end of an episode and the start of the next one) and predictable (episodes come at somewhat regular intervals over time)
- Infrequent and unpredictable (I don’t know when the next episode might come)
- Frequent (typically less than 3 months between the end of an episode and the start of the next one) and predictable
- Frequent and unpredictable
- Ongoing and occurs on most days
- I have only experienced one episode of [enter pain source] with no recurrence

**2. In the month when you most recently experienced [enter pain source], how often was the pain present?**

- Every day
- Most days
- Some days but not others
- Only a few days
- One day only

**3. On a typical day in the month when you most recently experienced [enter pain source], how would you describe the pattern of pain?**

- Continuous, steady, or constant
- Rhythmic, periodic, or intermittent
- Brief, momentary, or transient

**4. In the month when you most recently experienced [enter pain source], how would you rate the intensity of the pain at its worst?**

[No pain – 0 to 10 – Worst imaginable pain]

**5. In the month when you most recently experienced [enter pain source], how would you rate the average intensity of the pain?**

[No pain – 0 to 10 – Worst imaginable pain]

**6. In the month when you most recently experienced [enter pain source], how similar was the pain intensity across different episodes?**

- It was very similar or the same
- It differed a little bit across episodes
- It differed a lot across episodes

**7. In the month when you most recently experienced [enter pain source], during an average or typical day when you experienced [enter pain source], how much did it interfere with your ability to carry out your normal activities?**

- Not at all
- Very little
- Somewhat
- To a great extent

**8. Have you ever received medical care from your doctor or another health professional for [enter pain source]?**

- No
- Yes
  - If yes:
    - Please check all treatments you have received for [enter pain source]
      - Non-opioid analgesics (mono-substances, e.g. anti-inflammatories, acetaminophen, paracetamol, metamizol)
        - How effective was the treatment?

[Not at all – 0 to 10 – Completely ended my pain]

- - - - Weak and/or strong opioids
        - How effective was the treatment?

[Not at all – 0 to 10 – Completely ended my pain]

- - - - Prescribed medical marijuana (do not check if unprescribed)
        - How effective was the treatment?

[Not at all – 0 to 10 – Completely ended my pain]

- Rehabilitation
  - How effective was the treatment?

[Not at all – 0 to 10 – Completely ended my pain]

- Physiotherapy (physical therapy)
  - How effective was the treatment?

[Not at all – 0 to 10 – Completely ended my pain]

- Occupational therapy
  - How effective was the treatment?

[Not at all – 0 to 10 – Completely ended my pain]

- Exercise
  - - - - How effective was the treatment?

[Not at all – 0 to 10 – Completely ended my pain]

- - - - Surgery
        - How effective was the treatment?

[Not at all – 0 to 10 – Completely ended my pain]

- - - - Psychological or behavioural therapy
        - How effective was the treatment?

[Not at all – 0 to 10 – Completely ended my pain]

- - - - Other (If other, describe)
        - How effective was the treatment?

[Not at all – 0 to 10 – Completely ended my pain]

**9. Have you ever received alternative treatments outside of the healthcare system for [enter pain source]?**

- No
- Yes
  - If yes:
    - Vitamins, supplements, minerals, or herbs
    - Enzymes or hormones
    - Unprescribed medical marijuana
    - Mind-body exercises (e.g., meditation, relaxation)
    - Acupuncture
    - Massage
    - TENS (transcutaneous electrical nerve stimulation)
    - Other (If other, describe)

**10. Have you ever done things on your own to reduce [enter pain source]?**

- No
- Yes
  - If yes, please describe [textbox]

**11. Have you ever encountered barriers to improving pain management for [enter pain source]? If so, please describe them.**

- - - - No
      - Yes [textbox]

**Supplement 7: French Pre-NGT Survey**

**Enquête initiale - Étude sur les sources de douleur dans la sclérodermie**

**Évaluer l'importance de chacune des sources de douleur énumérées ci-dessous sur une échelle de 0 (pas du tout important) à 10 (extrêmement important).**

**Ensuite, le cas échéant, indiquez si vous pensez que cette source de douleur devrait être modifiée et expliquez pourquoi (par exemple, la description de la source de douleur n'est pas claire ou difficile à comprendre, ou la source de douleur est difficile à différencier des autres).**

**Source de douleur Modifier (si oui, expliquez pourquoi)** [oui, non]

**Ulcères sur les doigts ou les orteils**

[échelle de 0 à 10]

**Ulcères dans la bouche ou le nez**

[échelle de 0 à 10]

**Autres ulcères**

[échelle de 0 à 10]

**Gangrène des doigts ou des orteils (manque de circulation sanguine entraînant la mort des tissus corporels qui deviennent décolorés ou noirs)**

[échelle de 0 à 10]

**Amputation (liée à l'auto amputation)**

[échelle de 0 à 10]

**Douleur fantôme (due à la perte d'un doigt ou d'un orteil)**

[échelle de 0 à 10]

**Contractures des petites articulations (doigts, orteils)**

[échelle de 0 à 10]

**Contractures des grosses articulations (genoux, hanches, coudes, épaules)**

[échelle de 0 à 10]

**Phénomène de Raynaud**

[échelle de 0 à 10]

**Peau tendue, épaisse ou dure**

[échelle de 0 à 10]

**Calcinose (dépôt de sels de calcium insolubles dans les tissus cutanés et sous-cutanés, apparaissant généralement sous forme de bosses sous la peau)**

[échelle de 0 à 10]

**Syndrome du canal carpien (Trouble neurologique impliquant le nerf qui va de l'avant-bras à la paume de la main. Les symptômes comprennent l'engourdissement, le gonflement des doigts, les picotements, la perte de mouvement).**

[échelle de 0 à 10]

**Névralgie du trijumeau (douleur faciale aiguë ou fulgurante, soudaine et sévère)**

[échelle de 0 à 10]

**Symptômes gastro-intestinaux**

**Si oui:**

- **Déglutition**
- **Nausées**
- **Reflux acide ou brûlures d'estomac**
- **Ballonnements**
- **Constipation**
- **Diarrhée**

[échelle de 0 à 10]

**Oro-facial**

**Si oui:**

- **Ouverture de la bouche réduite**
- **Sécheresse due à une réduction de la production de salive**
- **Résorption ou érosion osseuse**
- **Indices de maladies parodontales (perte de tissu autour des dents, inflammation des gencives)**

[échelle de 0 à 10]

**Douleurs musculaires**

[échelle de 0 à 10]

**Douleur thoracique**

[échelle de 0 à 10]

**Douleurs d'origine autre que la sclérodermie**

**Si oui:**

- **Arthrose**
- **Douleurs dorsales**
- **Maux de tête**
- **Migraines**
- **Fibromyalgie (maladie chronique qui provoque des douleurs et une sensibilité dans tout le corps, de la fatigue et des troubles du sommeil)**
- **Autre [zone de texte]**

[échelle de 0 à 10]

**Pensez-vous qu'il manque des sources de douleur importantes dans la liste ci-dessus? Par exemple, y a-t-il un type de douleur que vous ressentez et qui n'a pas été mentionné?**

[oui, non]

Si oui, décrivez la source de douleur

**Évaluez l’importance de chaque série de questions énumérées ci-dessous sur une échelle de 0 (pas du tout important) à 10 (extrêmement important).**

**Ensuite, le cas échéant, indiquez si vous pensez que la question ou les options de réponse devraient être modifiées et expliquez pourquoi (par exemple, la question ou les options de réponse ne sont pas claires, compréhensibles ou pertinentes pour la sclérodermie).**

**Questions et réponses [échelle de 0 à 10] Modifier? (Si oui, expliquez pourquoi)** [oui, non]

**1. Depuis votre première expérience de douleur liée à [saisir la source de la douleur], les nouveaux épisodes ont été les suivants:**

- Peu fréquents (généralement plus de 3 mois entre la fin d'un épisode et le début du suivant) et prévisibles (les épisodes surviennent à intervalles assez réguliers au fil du temps)
- Peu fréquents et imprévisibles (je ne sais pas quand surviendra le prochain épisode)
- Fréquents (généralement moins de 3 mois entre la fin d'un épisode et le début du suivant) et prévisibles
- Fréquents et imprévisibles
- Permanent et se produit la plupart des jours
- Je n'ai connu qu'un seul épisode de [saisir la source de la douleur], sans récidive.

**2. Au cours du mois où vous avez ressenti le plus récemment une douleur [saisir la source de la douleur], à quelle fréquence la douleur était-elle présente?**

- Tous les jours
- Presque tous les jours
- Certains jours, mais pas d'autres
- Quelques jours seulement
- Un seul jour

**3. Au cours d'une journée typique du mois où vous avez ressenti le plus récemment une douleur [saisir la source de la douleur], comment décririez-vous l'intensité de la douleur?**

- Continue, régulière ou constante
- Rythmique, périodique ou intermittente
- Brève, momentanée ou transitoire

**4. Au cours du mois où vous avez ressenti le plus récemment une douleur [saisir la source de la douleur], comment évaluez-vous l'intensité de la douleur à son paroxysme?**

[Aucune douleur - 0 à 10 - Pire douleur imaginable]

**5. Au cours du mois où vous avez ressenti le plus récemment la douleur [saisir la source de la douleur], comment évaluez-vous l'intensité moyenne de la douleur?**

[Aucune douleur - 0 à 10 - Pire douleur imaginable]

**6. Au cours du mois où vous avez ressenti le plus récemment une douleur [saisir la source de la douleur], dans quelle mesure l'intensité de la douleur était-elle similaire d'un épisode à l'autre?**

- Elle était très similaire ou identique
- Elle différait un peu d'un épisode à l'autre
- Très différente d'un épisode à l'autre

**7. Au cours du mois où vous avez ressenti le plus récemment la douleur [saisir la source de la douleur], au cours d'une journée moyenne ou typique où vous avez ressenti la douleur [saisir la source de la douleur], dans quelle mesure cela a-t-il interféré avec votre capacité à mener vos activités normales?**

- Pas du tout
- Un peu
- Moyennement
- En grande partie

**8. Avez-vous déjà reçu des soins médicaux de votre médecin ou d'un autre professionnel de la santé pour [indiquer la source de la douleur] ?**

- Non
- Oui

o Si oui :

- Veuillez cocher tous les traitements que vous avez reçus pour [indiquer la source de la douleur]
- Analgésiques non opioïdes (mono-substances, par exemple anti-inflammatoires, acétaminophène, paracétamol, métamizol)

o Quelle a été l'efficacité du traitement ?

[Pas du tout - 0 à 10 - A complètement mis fin à ma douleur]

- Opioïdes faibles et/ou forts

o Quelle a été l'efficacité du traitement ?

[Pas du tout - 0 à 10 - A complètement mis fin à ma douleur]

- Cannabis médicale prescrit (ne pas cocher s’il n'est pas prescrit)

o Quelle a été l'efficacité du traitement ?

[Pas du tout - 0 à 10 - A complètement mis fin à ma douleur]

- Réhabilitation

o Quelle a été l'efficacité du traitement ?

[Pas du tout - 0 à 10 - A complètement mis fin à ma douleur]

- Physiothérapie (thérapie physique)

o Quelle a été l'efficacité du traitement ?

[Pas du tout - 0 à 10 - A complètement mis fin à ma douleur]

- Ergothérapie

o Quelle a été l'efficacité du traitement ?

[Pas du tout - 0 à 10 - A complètement mis fin à ma douleur]

- Exercice physique

o Quelle a été l'efficacité du traitement ?

[Pas du tout - 0 à 10 - A complètement mis fin à ma douleur]

- Chirurgie

o Quelle a été l'efficacité du traitement ?

[Pas du tout - 0 à 10 - A complètement mis fin à ma douleur]

- Thérapie psychologique ou comportementale

o Quelle a été l'efficacité du traitement ?

[Pas du tout - 0 à 10 - A complètement mis fin à ma douleur]

- Autre (Si autre, décrivez)

o Quelle a été l'efficacité du traitement ?

[Pas du tout - 0 à 10 - A complètement mis fin à ma douleur]

**9. Avez-vous déjà reçu des traitements alternatifs en dehors du système de santé pour une douleur liée à [saisir la source de la douleur]?**

- Non
- Oui
  - Si oui :
    - Vitamines, suppléments, minéraux ou plantes médicinales
    - Enzymes ou hormones
    - Cannabis médical non prescrit
    - Exercices psychocorporels (par exemple, méditation, relaxation)
    - Acupuncture
    - Massage
    - TENS (stimulation nerveuse électrique transcutanée)
    - Autre (si autre, décrivez)

**10. Avez-vous déjà fait quelque chose par vous-même pour réduire la douleur de [saisir la source de la douleur]?**

- Non
- Oui
  - Si oui, veuillez décrire [zone de texte]

**11. Avez-vous déjà rencontré des obstacles à l'amélioration de la prise en charge de la douleur pour [indiquer la source de la douleur] ? Si oui, veuillez les décrire.**

- Non
- Oui [zone de texte]

**Supplement 8: Follow-up Emails**

English Follow-up email

Subject: Reminder upcoming SPIN-PAIN group discussion

Dear [NAME],

This is a reminder that you are scheduled for an online group discussion on sources of pain in scleroderma on Month Date Year at TIME [XX:XX, XX AM/PM] TIME ZONE.

Please have your list of pain sources and questions that you believe should be removed, added, or modified readily available at the beginning of the group session. We suggest having a writing utensil and paper (or alternative device) available for brainstorming items during the session.

We kindly ask that you please come prepared to make sure the session runs smoothly.

**Zoom Instructions:**

On the day of your session, we suggest that you try to log on to *Zoom* at least 5 minutes before the start time to make sure you can comfortably use the program without any technical difficulties. Moderators will log into *Zoom* 15 minutes before the start of the session to help you resolve any technical issues if they were to arise.

Please let us know if you have any questions.

Kind regards,

The SPIN-PAIN Team

French Follow-up Email

Objet : Rappel à l’approche de la discussion du groupe SPIN-PAIN (étude sur la douleur)

Cher/chère [NOM],

Nous vous rappelons que vous êtes inscrit.e à une discussion de groupe en ligne sur les sources de douleur pour la sclérodermie qui se tiendra le mois date année à l'HEURE [XX:XX, XX AM/PM] FUSEAU HORAIRE.

Pour le début de la séance, veuillez avoir avec vous la liste des sources de douleur et des questions qui, selon vous, devraient être supprimées, ajoutées ou modifiées. Nous vous suggérons d'avoir à votre disposition un crayon et du papier (ou un autre moyen) pour réfléchir à des sujets pendant la séance.

Nous vous demandons de bien vouloir vous préparer à l’avance afin d'assurer le bon déroulement de la séance.

**Instructions pour Zoom:**

Le jour de votre séance, nous vous suggérons d'essayer de vous connecter à Zoom au moins 5 minutes avant l'heure de début afin de vous assurer que vous pourrez utiliser confortablement le programme sans rencontrer de difficultés techniques. Les modérateurs se connecteront à Zoom 15 minutes avant le début de la séance pour vous aider à résoudre d'éventuels problèmes techniques.

N'hésitez pas à nous faire part de vos questions.

Cordialement,

L'équipe SPIN-PAIN (étude sur la douleur)

**Supplement 9: Post-NGT Surveys**

**SPIN-PAIN ONLINE GROUP DISCUSSION**

**POST-SESSION SURVEY**

**You have participated in an online group session, during which you and other individuals discussed important sources of pain experienced in people with scleroderma.**

**Rate the importance of each of the pain sources listed below on a scale of 0 (not at all important) to 10 (extremely important).**

**Pain Source** [not at all important – 0 to – 10 – extremely important]

[Enter low rated pain sources discussed during the NGT session]

[Enter new pain sources discussed during the NGT session]

**Rate each set of questions listed below on a scale of 0 (not at all important) to 10 (extremely important).**

**Questions** [not at all important – 0 to – 10 – extremely important]

[Enter low rated questions]

[Enter new questions]

**DISCUSSION DE GROUP EN LIGNE SPIN-PAIN (ÉTUDE SUR LA DOULEUR)**

**ENQUÊTE POST-SÉANCE**

**Vous avez participé à une session de groupe en ligne, au cours de laquelle vous et d'autres personnes avez discuté des sources importantes de douleur ressenties par les personnes atteintes de sclérodermie.**

**Évaluez l'importance de chacune des sources de douleur énumérées ci-dessous sur une échelle de 0 (pas du tout importante) à 10 (extrêmement importante).**

**Source de douleur** [pas du tout importante - 0 à - 10 - extrêmement importante]

[Saisir les sources de douleur mal évaluées abordées au cours de la session NGT]

[Saisir les nouvelles sources de douleur abordées au cours de la session NGT]

**Évaluez chaque série de questions énumérées ci-dessous sur une échelle de 0 (pas du tout importante) à 10 (extrêmement importante).**

**Ensembles de questions** [pas du tout importante - 0 à - 10 - extrêmement importante]

[Saisir les séries de questions les moins bien notées]

[Saisir les nouvelles séries de questions]
